# Perinatal Outcomes of Small for Gestational Age in Twin Pregnancies: Twin vs. Singleton Charts

**DOI:** 10.3390/jcm10040643

**Published:** 2021-02-08

**Authors:** Veronica Giorgione, Corey Briffa, Carolina Di Fabrizio, Rohan Bhate, Asma Khalil

**Affiliations:** 1 Twins Trust Centre for Research and Clinical Excellence, St George’s University Hospitals NHS Foundation Trust, Blackshaw Road, London SW17 0RE, UK; giorgione.veronica@gmail.com (V.G.); m1305836@sgul.ac.uk (C.B.); carodifabrizio@gmail.com (C.D.F.); m1700399@sgul.ac.uk (R.B.); 2 Vascular Biology Research Centre, Molecular and Clinical Sciences Research Institute, St George’s University of London, Cranmer Terrace, London SW17 0RE, UK; 3 Fetal Medicine Unit, St George’s University Hospitals NHS Foundation Trust, Blackshaw Road, London SW17 0RE, UK

**Keywords:** twin pregnancy, singleton, estimated fetal weight, birth weight, small for gestational age, fetal growth restriction, chorionicity-specific, reference charts, ultrasound

## Abstract

Twin pregnancies are commonly assessed using singleton growth and birth weight reference charts. This practice has led to a significant number of twins labelled as small for gestational age (SGA), causing unnecessary interventions and increased risk of iatrogenic preterm birth. However, the use of twin-specific charts remains controversial. This study aims to assess whether twin-specific estimated fetal weight (EFW) and birth weight (BW) charts are more predictive of adverse outcomes compared to singleton charts. Centiles of EFW and BW were calculated using previously published singleton and twin charts. Categorical data were compared using Chi-square or McNemar tests. The study included 1740 twin pregnancies, with the following perinatal adverse outcomes recorded: perinatal death, preterm birth <34 weeks, hypertensive disorders of pregnancy (HDP) and admissions to the neonatal unit (NNU). Twin-specific charts identified prenatally and postnatally a smaller proportion of infants as SGA compared to singleton charts. However, twin charts showed a higher percentage of adverse neonatal outcomes in SGA infants than singleton charts. For example, perinatal death (SGA 7.2% vs. appropriate for gestational age (AGA) 2%, *p* < 0.0001), preterm birth <34 weeks (SGA 42.1% vs. AGA 16.4%, *p* < 0.0001), HDP (SGA 21.2% vs. AGA 13.5%, *p* = 0.015) and NNU admissions (SGA 69% vs. AGA 24%, *p* < 0.0001), when compared to singleton charts (perinatal death: SGA 2% vs. AGA 1%, *p* = 0.029), preterm birth <34 weeks: (SGA 20.6% vs. AGA 17.4%, *p* = 0.020), NNU admission: (SGA 34.5% vs. AGA 23.9%, *p* < 0.000). There was no significant association between HDP and SGA using the singleton charts (*p* = 0.696). In SGA infants, according to the twin charts, the incidence of abnormal umbilical artery Doppler was significantly more common than in SGA using the singleton chart (27.0% vs. 8.1%, *p* < 0.001). In conclusion, singleton charts misclassify a large number of twins as at risk of fetal growth restriction. The evidence suggests that the following twin-specific charts could reduce unnecessary medical interventions prenatally and postnatally.

## 1. Introduction

Twin gestation is a known risk factor for antenatal complications. However, increased fetal surveillance throughout twin pregnancies is associated with a lower risk of stillbirth [1,2,3]. An important element of twin surveillance includes monitoring to identify fetal growth restriction (FGR), as evidence suggests that growth-restricted infants are at increased risk of adverse perinatal outcomes, including increased mortality and morbidity [4]. As a result, it is vital to accurately assess the growth of twins antenatally and postnatally, to identify those who would benefit from preterm birth, altered surveillance and neonatal interventions.

It is currently a routine practice to assess both the antenatal growth and postnatal birthweight of twins using singleton estimated fetal weight (EFW) and birth weight (BW) charts, respectively. However, it has been shown that the use of singleton charts to monitor twin pregnancies is not accurate, as twins show a reduced growth rate in the third trimester [5]. As a result, a significant proportion of all twins are labelled as small for gestational age (SGA), leading to an increased risk of intervention and the subsequent risks associated with iatrogenic preterm birth [5,6,7]. Furthermore, evidence suggests that the median birth weight in twins is considerably lower than singletons from around week 30–32, which may be leading to unnecessary interventions neonatally [8,9,10].

To counter this, several twin-specific reference charts and BW reference charts have been suggested and created to help identify fetuses at risk of adverse outcomes [8,11,12,13,14]. However, their use remains controversial, as it is argued that the lower growth rates in twins may result from true placental insufficiency compared to singletons [15,16]. These pregnancies may be overlooked if twin-specific charts are implemented, potentially leading to increased morbidity and mortality rates.

Due to these two contrasting opinions, the validation of twin EFW and BW charts is crucial to understand their clinical impact before their use is routinely implemented. A previous study has suggested that while both customised and non-customised EFW singleton charts identify more SGA infants, they classify a similar proportion of stillborn infants compared to the twin-specific EFW charts. Therefore, introducing the twin charts can reduce unnecessary medical intervention [7]. Consequently, it would be wise to perform a similar analysis of twin-specific BW charts and compare more recent singleton fetal growth and BW charts like those presented by Nicolaides et al. [17].

This study’s main aim was to assess if chorionicity-specific twin growth and birth weight reference charts can better identify high-risk pregnancies compared to previously published EFW and BW charts—evaluating their ability to predict adverse fetal and neonatal outcomes, including perinatal death, preterm birth (PTB), hypertensive disorders of pregnancy (HDP) and admission to the neonatal unit (NNU).

## 2. Materials and Methods

### 2.1. Study Population

A retrospective cohort study of prospectively collected data was performed. The inclusion criteria were unselected twin pregnancies at St George’s Hospital, London, UK, between January 2000 and May 2020. Pregnancies were identified by searching the electronic maternity records (ViewPoint version 5.6.26.148, ViewPoint Bildverarbeitung GMBH, Wessling, Germany) and pregnancy outcomes were established from the maternity database and neonatal records. Exclusion criteria included significant fetal structural anomalies or aneuploidy, intrauterine death (IUD) of one twin before 24 weeks’ gestation, miscarriage and missing gestational age at delivery.

### 2.2. Study Variables and Outcomes

Data on maternal age in years, body mass index (BMI) in kg/m^2^, reported ethnicity, mode of conception and smoking were collected. The last prenatal ultrasound examination reporting EFW and fetal Doppler assessment were used in the analysis. HDP included gestational hypertension and preeclampsia defined by the International Society for the Study of Hypertension in Pregnancy (ISSHP) guidelines [18]. Chorionicity was determined based on the presence or absence of the lambda sign at the intertwin membrane–placenta junction, as well as the intertwin membrane thickness at the site of its insertion in the chorion at 11–14 weeks, or the number of placentas and the fetal gender after 14 weeks’ gestation [19,20,21]. Gestational age (GA) was determined according to the crown-rump length (in the first trimester) or head circumference (after 14 weeks’ gestation) of the larger fetus in cases of spontaneous conception and according to the embryonic age from fertilisation if in-vitro fertilisation had taken place [19,22,23]. The centiles of EFW were calculated adopting the singleton chart by Nicolaides et al. [17] and the twin charts by Stirrup et al. [9]. The BW centiles were assessed using the singleton standard reported by Nicolaides et al. [17] and twin chorionicity-specific reference standards reported by Briffa et al. [24]. Fetuses with EFW less than 10th centile and newborns with BW less than 10th centile were considered SGA. The outcomes of SGA fetuses, according to twin charts, were compared with those of SGA fetuses according to singleton charts. The same comparison was performed among SGA neonates defined by twin or singleton standards. Moreover, a sub-group analysis was performed in SGA twins identified by both charts, SGA twins identified only by singleton charts and in AGA infants detected by both charts before birth (using EFW) and after birth (using BW).

The study outcomes were:

- Perinatal death, which includes stillbirth (IUD after 24 completed weeks of pregnancy) and early neonatal death (death of a newborn within seven days after birth),
- PTB before 34 weeks’ gestation,
- HDP,
- Admission to NNU.

We also evaluated fetal Doppler abnormalities in the twin pregnancies with SGA fetuses defined by twin vs. those by singleton charts. The pulsatility index (PI) of the umbilical artery (UA) and middle cerebral artery (MCA) and the cerebro-placental ratio (CPR), which is the ratio between MCA-PI and UA-PI, were recorded. Abnormal UA Doppler was defined when the PI was above the 95th centile [25], absent (AEDF) or reversed EDF (REDF).

### 2.3. Statistical Analysis

Categorical data were presented as number (%) and compared using Fisher’s exact test or Chi-square test. The D’Agostino and Pearson Omnibus test was used to assess the normality of the data. Continuous data were presented as median (interquartile range, IQR).

According to twin and singleton charts, the proportions of SGA fetuses and neonates were determined and compared using Chi-squared and McNemar tests. A logistic regression model was performed in univariate analysis to study the association between adverse outcomes and growth abnormalities in twins identified by using different standards during pregnancy and at birth. Odds ratio (OR) and 95% confidence intervals (95% CI) were calculated. *p*-values below 0.05 were considered statistically significant. The statistical analysis was performed using SPSS version 26.0 (SPSS, Statistical Package for the Social Sciences, IBM, Armonk, NY, USA) statistical software.

## 3. Results

### 3.1. Study Population

The study included 1740 twin pregnancies: dichorionic (77.5%) and monochorionic (22.5%). The median maternal age was 34 years, and median BMI was 24.4 kg/m^2^. Data on demographics, pregnancy characteristics and outcomes are shown in Table 1. The median gestational age at delivery was 36.7 weeks and the incidence of PTB before 34 weeks’ gestation was 20.3%. The incidence of HDP was 14%, while that of perinatal death was 1.5%. The incidence of NNU admission was 29.1%.

Using the twin chart, 278 fetuses (8.3%) were identified as SGA, while the singleton chart identified 1172 SGA fetuses (34.8%) (*p* < 0.001). Number of neonates with a BW less than 10th centile were 249 (7.3%) using the twin chart and 1324 (38.4%) using the singleton chart (*p* < 0.001).

### 3.2. EFW Centile According to Twin References versus Singleton References

EFW less than 10th centile using a twin chart was significantly associated with the risk of perinatal death (7.2% vs. 0.8%, *p* < 0.0001, OR 9.14, 95% CI 5.0–16.6, *p* < 0.0001). Similarly, the risk of PTB prior to 34 weeks’ gestation was significantly higher than in fetuses classified as AGA (42.1% vs. 16.4%, *p* < 0.0001, OR 3.7, 95% CI 2.9–4.8, *p* < 0.0001). The incidence of HDP was also significantly higher in SGA than AGA fetuses (21.2% vs. 13.5%, *p* = 0.015, OR 1.7, 95% CI 1.1–2.7, *p* = 0.016). The risk of NNU admission was also significantly higher in SGA than in AGA fetuses (69% vs. 24%, *p* < 0.0001, OR 7.1, 95% CI 5.4–9.3, *p* < 0.0001) (Table 2 and Table 3).

EFW less than 10th centile using a singleton chart was significantly associated with the risk of adverse outcomes (perinatal deaths: 2% vs. 1%, *p* = 0.029, OR 1.9, 95% CI 1.1–3.4, *p* = 0.032; PTB prior to 34 weeks: 20.6% vs. 17.4%, *p* = 0.020, OR 1.2, 95% CI 1.0–1.5, *p* = 0.020; NNU admission: 34.5% vs. 23.9%, *p* < 0.0001, OR 1.7, 95% CI 1.4–2.0, *p* < 0.0001). The strength of association was lower than when using the twin chart. There was no significant association between HDP and SGA using the singleton charts (*p* = 0.696) (Table 2 and Table 3).

When each adverse perinatal outcome in SGA babies was compared using the different charts, SGA babies defined by the twin references were more frequently associated with perinatal death (7.2% vs. 2%, *p* < 0.0001), PTB before 34 weeks’ gestation (42.1% vs. 20.6%, *p* < 0.0001) and NNU admission (69% vs. 34.5%, *p* < 0.0001).

**Figure 1:**
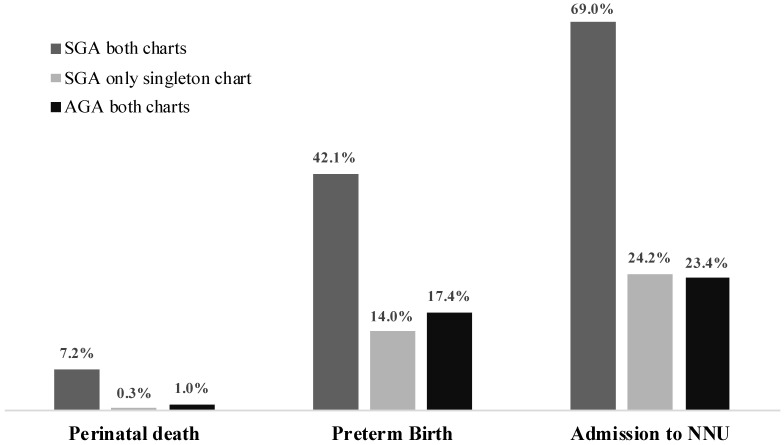
Adverse perinatal outcomes in small for gestational age (SGA) fetuses according to singleton and twin charts, SGA identified only by singleton chart and non-SGA (AGA) fetuses according to singleton and twin charts using estimated fetal weight.

Figure 1 illustrates the frequency of adverse fetal outcomes in SGA twins detected by both EFW charts, in SGA twins identified by only singleton charts and AGA according to both EFW charts. The proportions of perinatal death, PTB and NNU were similar among SGA determined by only singleton charts and AGA according to both singleton and twin EFW charts.

### 3.3. The Comparison of Doppler Assessment in Fetuses with EFW Less than 10th Centile According to Twin vs. Singleton Chart

Doppler assessments were available for 1132 out of 1172 fetuses defined as SGA using the singleton chart and 267 out 278 SGA using the twin chart. The median (IQR) UA-PI was 1.13 (1.00–1.46) in SGA fetuses defined by the twin chart and 1.01 (0.88–1.15) in those defined by singleton chart, while the median (IQR) CPR was 1.25 (0.88–1.75) in the former group and 1.62 (1.86–1.41) in the latter group. According to the twin charts, in fetuses with EFW < 10th centile, the incidence of abnormal UA Doppler, including UA-PI above the 95th centile AEDF or REDF, was significantly more common than in SGA using the singleton chart (27.0% vs. 8.1%, *p < 0.001*).

### 3.4. BW Centile According to Twin vs. Singleton Chart

Compared to neonates with a BW more than 10th centile, SGA neonates defined by the twin chart were significantly more likely to experience perinatal death (3.2% vs. 0.4%, OR 8.0, 95% CI 3.3–19.5, *p* < 0.0001), PTB prior to 34 weeks (36.9% vs. 18.1%, OR 2.7, 95% CI 2.0–3.5, *p* < 0.0001), NNU admission (66.3% vs. 25.3%, OR 5.6, 95% CI 4.3–7.4, *p* < 0.0001) and HDP (13.5% vs. 23.5%, OR 2.0, 95% 1.2–3.2, *p* = 0.005). Conversely, no significant differences were found when the singleton chart was used to define SGA newborns (Table 2 and Table 3).

**Figure 2:**
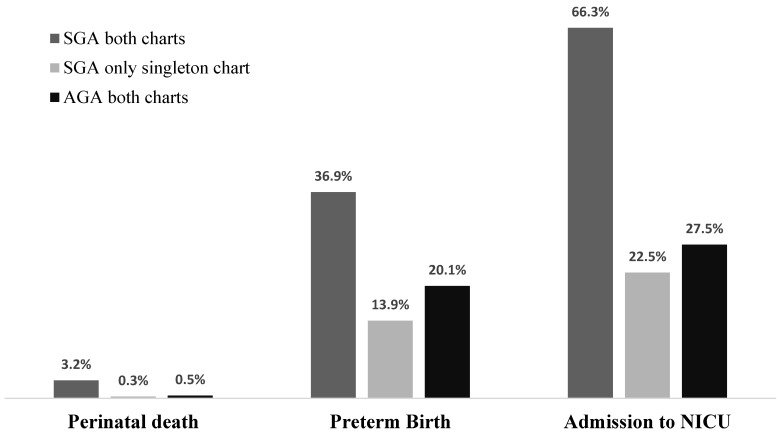
Adverse perinatal outcomes in small for gestational age (SGA) fetuses according to singleton and twin charts, SGA identified only by singleton chart and non-SGA (AGA) fetuses according to singleton and twin charts using birth weight.

Figure 2 illustrates the frequency of adverse outcomes in SGA twins detected by both BW charts, in SGA twins identified by only singleton charts and AGA according to both charts. The proportions of perinatal death, PTB and NNU were similar among SGA identified by only singleton charts and AGA according to both singleton and twin charts.

## 4. Discussion

### 4.1. Summary of Main Findings

Twin-specific charts identified prenatally and postnatally a smaller proportion of fetuses or neonates as SGA than singleton EFW and BW charts. However, of those detected as SGA using twin-specific references, the likelihood of those infants developing adverse neonatal outcomes was significantly higher than those labelled as SGA on the singleton reference chart alone. Finally, abnormal fetal Doppler was more common in the SGA defined using the twin than the singleton charts.

### 4.2. Interpretation of Study Findings and Comparison with Published Literature

Using customised growth charts according to pregnancy-specific variables has been a controversial subject [26]. However, the evidence suggests that their use can improve our ability to identify fetuses at risk of adverse outcomes. Adjusting for twin-specific variables, for example, chorionicity should, in theory, improve the ‘one-size-fits-all’ approach currently offered by the use of singleton charts [7]. Recent studies have also proven that adjusted EFW reference charts for twins reduce the number of twins labelled as SGA and still identify a similar proportion of stillbirth cases [7]. Furthermore, published studies have highlighted that twin-specific birthweight charts were more strongly associated with HDP and PTB than the singleton charts [27,28,29]. Our results reveal a similar trend, suggesting that SGA defined using twin-specific charts, both EFW and BW, had a stronger association between those labelled as SGA and those at risk of adverse outcomes. The fact that the finding of an abnormal fetal Doppler was more common in the SGA fetuses defined using twin charts than those using singleton charts suggests the use of twin charts to identify the fetuses suffering from growth restriction, and hence, at risk of adverse perinatal outcomes. We previously reported that the combination of discordance in fetal size and Doppler waveform can predict the risk of perinatal loss in twin pregnancies [25].

### 4.3. Clinical and Research Implications

Twin pregnancies are monitored frequently due to their high risk of antenatal and perinatal complications [19,30,31]. However, current methods to assess twin pregnancies using singleton charts are inadequate. The literature suggests that the growth trajectory of twin pregnancies from 30 weeks onwards is reduced compared to singletons. As a result, a significant proportion of twin pregnancies are classified as SGA [5]. Therefore, as previously described, the rationale behind the development of twin-specific charts is to account for this difference in growth rates and reduce the number of twins identified as SGA, reducing unnecessary interventions, in particular, iatrogenic prematurity, NNU admission and long-term risk of prematurity-related disability [7].

Our study suggests that using twin-specific EFW and BW charts significantly reduces the proportion of twins classified as SGA. Moreover, these twin-specific charts show a significant increase in the proportion of SGA infants who experience an adverse outcome, thereby suggesting that the twin charts are more specific for identifying these high-risk pregnancies. The benefit of using twin-specific reference charts prenatally is their ability to reduce the proportion of twins identified as SGA, helping to minimise the risk associated with preterm iatrogenic birth and the developmental complications associated with it. In addition to this, our study found that twin-specific BW charts are identifying SGA twins who are associated with a greater risk of adverse outcomes. These BW charts will allow clinicians to take a more specific approach to smaller infants, potentially helping to dictate neonatal management of those infants who are genuinely growth restricted, reducing the burden of NNU admissions and reducing the cost of care.

Our study provides initial evidence that supports the safety of twin-specific growth and birth weight charts. However, it would be prudent to recommend prospective and extensive multi-centre studies.

### 4.4. Strength and Limitations

Our study’s strengths include the use of a large and diverse cohort of twin pregnancies from a tertiary care centre in London. The use of modern growth charts, which were not in active clinical use in our population, will help reduce the risk of intervention bias. The singleton charts opted for use in this study were recent and derived from a cohort size of 95,579 pregnancies, all of which used EFW to derive their BW charts. Additionally, the singleton charts were from a population followed up at a tertiary care centre in London and a population similar to our cohort. We built on the previous work of Kalafat et al. [7] to include a comparison of the most recent twin and singleton reference charts.

Furthermore, we examined the Doppler assessment, a well-defined marker of fetal growth restriction, in our cohort. However, it should be noted this was a retrospective analysis of routinely collected data and that the number of perinatal deaths in our cohort was small. Therefore the analysis for the detection of perinatal death could be underpowered. Finally, the twin BW charts used were developed from some pregnancies in this cohort, which may lead to an overestimation of the twin BW chart’s performance. However, as these BW charts were not in routine clinical use, it is unlikely to represent an intervention bias risk.

We have not assessed all potential confounding factors, for example, gestational and pregestational diabetes due to missing data. However, we expect this number to be small in this population, reducing the risk of a confounding bias. Finally, this data’s generalizability should be cautioned as the results found are only specific to the population cohort assessed, therefore further prospective studies are recommended before implementation is considered.

## 5. Conclusions

Compared to the twin-specific growth and BW charts, singleton charts classified more fetuses as SGA prenatally and more neonates as SGA postnatally. However, the twin-specific charts used in the study identified a higher proportion of SGA fetuses who had adverse outcomes and more fetuses with growth restriction as indicated by abnormal fetal Doppler assessment. This evidence suggests that the twin-specific charts can safely reduce unnecessary medical interventions prenatally and postnatally.

## Figures and Tables

**Table 1:** Baseline demographics and pregnancy characteristics of the study cohort.

	Twin Pregnancies (*n* = 1740)
Maternal age in years, median (IQR)	34 (30–37)
Maternal body mass index in kg/m^2^, median (IQR)	24.4 (21.8–27.9)
Nulliparous, *n* (%)	975 (56)
Racial origin, *n* (%)	
▪ Caucasian ▪ Afro-Caribbean ▪ Asian ▪ Mixed ▪ Others	1122 (64.8) 279 (16.1) 205 (11.8) 29 (1.7) 97 (5.6)
Smokers, *n* (%)	89 (5.2)
Assisted conception, *n* (%)	477 (27.6)
Pre-existing hypertension, *n* (%)	59 (3.4)
Gestational age at delivery in weeks, median (IQR)	36.7 (34.6–37.4)
Hypertensive disorders of pregnancy, *n* (%)	243 (14)
Preterm birth before 34 weeks, *n* (%)	354 (20.3)
Twin-to-twin transfusion syndrome, *n* (%)	54 (3.1)
	**Twins** **(*n* = 3480)**
Pregnancy outcome	
▪ Stillbirth, *n* (%) ▪ Early neonatal death, *n* (%)	31 (0.9) 22 (0.6)
Birthweight in grams, median (IQR)	2301 (1902–2595)
Admission to the neonatal unit, *n* (%)	1003 (29.1)

**Table 2:** Adverse perinatal outcomes in small for gestational age (SGA) fetuses according to twin vs. singleton chart.

Adverse Outcomes	EFW-Twin Chart			EFW-Singleton Chart			BW-Twin Chart			BW-Singleton Chart		
	SGA	AGA	*p* Value *	SGA	AGA	*p* Value *	SGA	AGA	*p* Value *	SGA	AGA	*p* Value *
Perinatal death, *n* (%)	20 (7.2)	26 (0.8)	<0.0001	23 (2)	23 (1)	0.029	8 (3.2)	13 (0.4)	<0.0001	11 (0.8)	11 (0.5)	0.263
Preterm birth <34 weeks, *n* (%)	117 (42.1)	507 (16.4)	<0.0001	242 (20.6)	282 (17.4)	0.020	92 (36.9)	567 (18.1)	<0.0001	242 (18.3)	442 (20.8)	0.067
NNU admission, *n* (%)	185 (69)	736 (24)	<0.0001	401 (34.5)	520 (23.9)	<0.0001	165 (66.3)	811 (25.8)	<0.0001	406 (30.7)	593 (28)	0.095
HDP, *n* (%)	28 (21.2)	210 (13.5)	0.015	152 (13.9)	86 (14.6)	0.696	24 (23.5)	215 (13.5)	0.005	97 (15.1)	143 (13.2)	0.273

* The results of Chi-square test are presented.

**Table 3:** The association between small for gestational age (SGA) and adverse perinatal outcomes according to whether SGA was defined using the twin vs. singleton chart.

Adverse Outcomes	Estimated Fetal Weight < 10th Centile					
	Twin Chart			Singleton Chart		
	OR	95% CI	*p* Value	OR	95% CI	*p* Value
Perinatal death	9.14	5.0–16.6	<0.0001	1.9	1.1–3.4	0.032
Preterm birth prior to 34 weeks	3.7	2.9–4.8	<0.0001	1.2	1.0–1.5	0.020
Neonatal unit admission	7.1	5.4–9.3	<0.0001	1.7	1.4–2.0	<0.0001
Hypertensive disorders of pregnancy	1.7	1.1–2.7	0.016	1.1	0.8–1.4	0.696
	**Birth Weight < 10th Centile**					
	**Twin Chart**			**Singleton Chart**		
	**OR**	**95% CI**	***p* Value**	**OR**	**95% CI**	***p* Value**
Perinatal death	8.0	3.3–19.5	<0.0001	1.6	0.7–3.7	0.268
Preterm birth prior to 34 weeks	2.7	2.0–3.5	<0.0001	0.9	0.7–1.0	0.067
Neonatal unit admission	5.6	4.3–7.4	<0.0001	1.1	1.0–1.3	0.095
Hypertensive disorders of pregnancy	2.0	1.2–3.2	0.006	1.2	0.9–1.5	0.274

## Data Availability

The data presented in this study are available on request from the corresponding author. The data are not publicly available to due to privacy reasons.
